# Knowledge, attitude and practices related to tuberculosis among patients at the Presbyterian Hospital in the Asante Akim North District

**DOI:** 10.4314/ahs.v24i2.10

**Published:** 2024-06

**Authors:** Sebastian Osei Kwarteng, Eric Sampane Donkor, Julius Eyiuche Nweze

**Affiliations:** 1 School of Public Health, College of Health Sciences, Kwame Nkrumah University of Science and Technology, Kumasi, Ghana; 2 Department of Biological Chemistry, Institute for Organic Chemistry, Faculty of Engineering and Natural Sciences, Johannes Kepler University — Linz, Austria; 3 Faculty of Sciences, University of South Bohemia, České Budějovice, Czech Republic; 4 Laboratory of Molecular Biology of Ticks, Institute of Parasitology, Biology Centre, CAS, CeskeBudejovice, Czech Republic; 5 Department of Medical Microbiology, University of Ghana Medical School, Accra, Ghana; 6 Department of Ecosystem Biology, Faculty of Science, University of South Bohemia in Ceske Budejovice, Czech Republic; 7 Soil and Water Research Infrastructure, and Institute of Soil Biology, Biology Centre, CAS, Česke Budejovice, Czech Republic

**Keywords:** Knowledge, attitude, practices, tuberculosis, Presbyterian Hospital in the Asante Akim North District

## Abstract

**Background:**

Tuberculosis (TB) is one of the major public health concerns in Ghana, with serious economic and social consequences. Tuberculosis is an infectious disease that is preventable and curable, as health educational programmes contribute to the control of TB However, the evidence required for such programmes is lacking in Ghana.

**Objectives:**

The aim of this study was to examine the underlying practices, attitudes and knowledge (PAK) of the patients at the Presbyterian hospital in Agogo, the Asante Akim North District (PHAA-AND) about tuberculosis disease and healthcare-seeking behaviour.

**Methods:**

This was a cross-sectional study among patients in the PHAA-AND. A simple random sampling method was used in selecting 370 participants for the study, who were interviewed regarding their TB knowledge, attitude and infection control practices.

**Results:**

Our study shows that the majority of the respondents demonstrated good knowledge about TB regarding its causative agent (68%), transmission (85.6%) and prevention (81.7%). However, poor knowledge was expressed regarding TB treatment by the majority (80.8%) of the respondents. Generally, the majority of respondents had a positive attitude and expressed good infection control practices regarding TB. The strongest determinants of TB related knowledge, or attitude or towards infection control practices were; level of education (OR, 1.49, CI; 1.25-1.77, p < 0.001), and gender (OR, 0.37, CI; 0.21-0.69, p=0.001).

**Conclusions:**

Respondents had good PAK towards TB, though some gaps were identified. These gaps called for health education about TB in the study area, and effective educational programs.

## Introduction

Tuberculosis (TB) is a disease of global public health importance, with the global agenda to end it by 2030^1^. It is one of the primary causes of death among HIV-positive patients, as well as a significant contributor to antibiotic resistance.^2^ According to the Global Tuberculosis 2019 Report, 10 million people contract tuberculosis each year, 3 million of them go untreated or unreported, and 1.5 million people die as a result of the disease.^3^ Overall, there was a 21% decrease in TB notifications between 2019 and 2020, with substantially higher reductions in some countries with high TB burdens.^4^ More than a third of these cases, however, might have been missed by health services and remain undiscovered^5^ undiscovered countries, especially those Countries with high TB burdens, are not on course to reach this long-term development goal. Individual awareness has an impact on TB transmission and early TB screenings, which could assist to halt the TB epidemic. Apart from health related issues, TB has a wide range of consequences, including serious economic crises in developing countries.^6^

Tuberculosis is principally caused by Mycobacterium tuberculosis, a bacterium that often affects the lungs. According to a study conducted in Ghana, the majority of tuberculosis cases (97.6%) are caused by Mycobacterium tuberculosis, whereas 2.4 percent are caused by Mycobacterium africanum^7^,^8^ mycobacterium africanum. It is spread mainly through air from an infected person and/or place, primarily through coughing, sneezing, or spitting by persons with lung TB^9^ TB. The signs of tuberculosis may include coughing, night sweats, fever, loss of weight and loss of appetite.^10^ Majority of persons who contract tuberculosis live in low- and middle-income nations, but the disease is found all around the world. China, the Philippines, Bangladesh, Indonesia, India, Pakistan, and some Africa countries account for roughly half of all persons with tuberculosis.^3^ A minimum of six months of treatment is required for TB. Patients may not be cured if treatment is not completed, and drug resistance may develop. Directly Observed Therapy (DOT) is a World Health Organization-approved technique for improving adherence by trained health staff, community volunteers, or family members to monitor and document patients as they take each dose.^11^ Without proper information and treatment, an infected person can infect 10-15 people every year. The current situation of TB management in Ghana and some other developing countries requires attention. While Ghana is not part of the high burden countries, recorded cases of tuberculosis advise that continuous monitoring of the disease is necessary to prevent abrupt widespread.^3^ According to the Ghana Health Service, 286 out of 100,000 people in Ghana are infected with TB annually. Data from the Ghana Country Coordinating Mechanism (CCM) of the Global Fund showed the estimated number of people who contract TB decreased from 45,000 in 2015 to 45,000 in 2019 where about 14,691 infected people successfully treated acquired treatment. The cases of TB-HIV co-infection and drug resistant TB increased from 8600 and 870 in 2018 to 9200 and 1200 in 2019 respectively. Progression from latent TB to active TB could be contributed by poor socio-economic position status, poor nutritional status, HIV infection, and smoking. Low socio-economic status, lack of access to health care, and lack of understanding about the disease and its way of transmission are all determinants in contracting TB.^12^ Moreover, the scarcity of information, attitude, and beliefs that arise as a result of wrong information about tuberculosis is a major challenge for controlling the disease spread. This often leads to delayed health-seeking and stigmatization of infected persons. To the best of our knowledge, there are currently no data on the PAK about tuberculosis in Ghana. As a result, the study was carried out to examine the PAK among patients visiting PHAA-AND, about tuberculosis disease and healthcare seeking behaviour.

## Materials and Methods

### Study setting and design

The study was a descriptive cross-sectional survey of PAK related to TB among patients. The study was done at the PHAA-AND, which is a multi-ethnic with all three main religions in Ghana present (i.e. Christianity, Islam and Traditional religions). Farming is the main economic activity in this area. The district is served by two health centres, Juansa Health Center at Juansa and Presbyterian Hospital in Agogo, which has a TB facility. Agogo was chosen purposely because of the rising number of tuberculosis cases annually and the fact that it has a TB management centre. The TB management centre had a total of 155 and 157 tuberculosis cases under treatment in 2017 and 2018, respectively.

### Target population and sampling

The target population were both outpatients and inpatients at the PHAA-AND. The simple random sampling method was used to provide equal opportunity for everyone to be selected for the study.

### Inclusion and exclusion criteria

All clients of the age 20 and above visiting the outpatient and inpatients departments at the Presbyterian Hospital in Agogo during the data collection time were included. The respondents had to be residents in the district for at least five months. Clients who were severely sick, and healthcare workers (HCW) in the health facility, didn't participate in the trial.

### Research instruments and data collection

A well-designed open-ended questionnaire was used to gather data from the study participants. The questionnaire was pretested among twenty people who were within the sampling plan to ensure its validity and reliability. The research instrument covered five areas including demographic data, PAK of tuberculosis, and healthcare seeking behaviour. The development of the research instruments was based on review of relevant literature. The questionnaire was translated into Twi language as it is the local and widely spoken language in the study area. TB knowledge was assessed using these knowledge assessment variables: the cause, transmission mode, signs and symptoms, prevention, where to seek healthcare, curability, drug availability, treatment cost, drugs cost, and duration of treatment. For attitude towards TB, it was assessed based on people's perception towards infected persons, infectability of TB, and concerns of HIV-positive patients about TB. In the case of practices towards tuberculosis, ie what people do to control it and how often do they seek medical care were the variables of assessment. Healthcare seeking behaviour and practices on treatment of TB were also assessed based on where people go for treatment, how often people usually sought healthcare at clinics or hospitals, what people do if they detect symptoms of TB, and at what point they'll visit the health facility if they had symptoms of TB.

PAK and healthcare behaviour scores on each of these variables were generated. Correct/appropriate responses were based on literature and best practices, and were scored a “1”, while incorrect/inappropriate responses were scored “0”.

### Data processing and analysis

The sample size for the study was calculated using the formula proposed by^13^. It is based on the following assumptions: 95% confidence interval (Z), 5% margin of error (e), estimated prevalence of TB is 50% (P), and a total sample of 370 was needed (N).

### Sample size


$$\frac{\frac{z^{2}\cdot p(1-p)}{e^{2}}}{1+\left(\frac{z^{2}\cdot p(1-p)}{e^{2}N}\right)}$$

Consistency and completeness of data gathered was checked. MS-EXCEL was used in coding and entering the data, while STATA V14 and R were used to analyse the data.

### Ethical consideration

Ethical clearance was sought from the KNUST ethical clearance committee (CHRPE) for the study. Informed consent was sought from the study participants. Confidentiality and privacy of participants were respected and protected by not including names in the data entry. All the data gathered from the participants was used for research purpose only.

## Results

### Demographic characteristics of study participants

**Figure 1:**
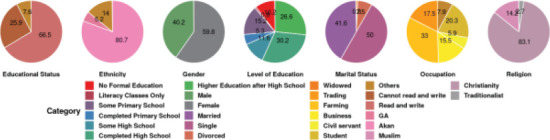
Demographic features of the respondents

Three hundred and seventy people participated in the study and their demographic features were recorded (Fig. 1). The majority of the study participants, constituting 30.3% (109) had completed high school while 26.6% (96) had completed higher education after high school. It was further realized that 10.2 % (37) had never been to school, whiles 0.8% (3) attended literacy classes. In terms of gender, most of the participants were females (219) representing 59.9% of the participants, while males formed 40.1%. A proportion of 50% (179) of the study participants were unmarried (single), 41.7% (149) were married, 7.4% (27) were divorced, while 0.8% (3) were widowed. In terms of occupation, most of the participants were farmers (117) constituting 32.9%, followed by students who constituted 20.4% (72) of the respondents. A huge majority of the participants were from the Akan tribe (80.8%) and were Christians (83.1%).

### Demographic features of the respondents

#### Knowledge of TB of the respondents

Two hundred and forty-six respondents, representing 68%, mentioned that bacteria are the cause of TB. In terms of seriousness of TB disease, the majority of the participants, representing 85.6% pointed out that the disease is very serious (Table 1).

**Table 4.2.1:** Knowledge Level of the Participants on Tuberculosis

Knowledge	Percentage score for correct Answer
**Cause of TB:**	
Bacteria	68% (246)
Seriousness of TB: Very serious	85.6% (317)
**Signs and symptoms of Tuberculosis:**	
Cough	50.6% (185)
Severe headache	16.2% (59)
Nausea	13.8% (51)
Weight loss	44.1% (161)
Fever without clear cause	7.3% (27)
Chest pains	28.3% (104)
Shortness of breadth	13% (48)
**Transmission of TB**	
Through the air	35.6%(317)
**Prevention of TB**	
Covering of mouth and nose when coughing	81.7% (284)
Avoid eating of raw meat	17.9% (62)
Avoid drinking of raw milk	17.4% (61)
**Who can be infected with Tuberculosis**	
Anybody	92.2% (335)
**HIV patient should be concerned about TB**	75% (271)
**TB can be cured**	96.5% (327)
**Treatment of TB**	
DOTS	19.2% (65)
Specific drugs given by health centre	83.4% (297)
Drugs available for free	41.4% (145)

Source: Authors field survey data compilation, 2018.

On the signs and symptoms, 50.6% identified that cough is a sign and symptom of TB, while 44.1% identified weight loss. A high majority of the study participants (85.6%) indicated that TB can be contracted through air. Regarding knowledge on TB prevention, the majority of the respondents (81.7%) indicated TB can be prevented by covering mouth and nose when coughing. However, minority of the respondents indicated that TB can be prevented by avoiding eating raw meat and raw milk (Table 1). The results further indicated that, greater number of the participants (92.2%) have the knowledge that anybody can be infected with TB disease. Also, the majority of the respondents have the knowledge that HIV patients should be concerned with TB disease (75%) and that TB is curable (96.5%). On the treatment of TB, only 19.2% of the respondents were aware of DOTS. However, the majority of them, indicated that TB can be treated through drugs provided by the health centre. A proportion of 41.4% of the study participants indicated that drugs are available for free for the treatment of TB.

#### The attitudes towards tuberculosis

**Fig. 2:**
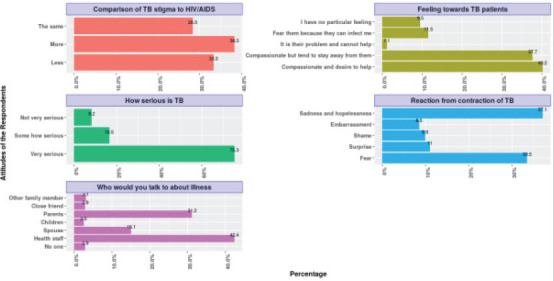
Attitudes of the Respondents towards TB and TB Patients

Regarding the attitude and perception towards TB infection (Fig. 2), Most of the respondents showed a positive attitude by responding that TB is very serious, while 16.5% (56) indicated TB is somehow serious). The minority of them indicated that TB is not very serious. The study also identified the feelings that the respondents have towards those who have TB disease. Less than half of the respondents (40.1%, n = 144) are compassionate about TB patients and are willing to help them, while the remainder of the study participants avoided TB patients. In terms of the respondents' reaction to the contraction of TB disease, 49.8%(179) of the respondents indicated sadness and hopelessness, 44.9% (162) indicated fear, while 14.8% (53), 13.2% (48) and 11.5% (41) indicated surprise, shame and embarrassment respectively. As to who the respondents will report to when they contract TB disease, the majority of them indicated they would report to health staff. About 36.2 % (130) of the respondents indicated that TB disease carry more stigma than HIV, 31.3%indicated that TB disease carry less stigma than HIV/AIDS, while 26.7% indicated that TB disease has the same stigma as the HIV/AIDS.

#### Practices towards the prevention of tuberculosis

Regarding practices towards TB prevention and control (Table 2, 81.7% of the study participants expressed good practice by indicating one must cover the mouth and nose when coughing). On the frequency of medical check-up for TB and other related infections, 17.9% indicated twice or more in a year, 17.0% once every year, 20.2% once every two years, 5.8% once or twice in five years, 19.3% never in the last five years while 19.7% indicated that they do not attend any checkup at all. The study also sought to find out the point at which respondents would seek help from a health centre when they realized symptoms of TB. In response to this,70.6% of them indicated that immediately they realize signs and symptoms, 17.7% indicated when self-treatment fails, 6% (22) when the signs and symptoms have lasted for 3 to 4 weeks while 5.6% (21) mentioned that they would not go to the health centre at all.

**Table 4.2.3:** Practices towards the Prevention and Control of Tuberculosis

Practices	Response	Frequency	Percentage
What to do when sneezing and coughing	Cover nose and mouth	284	81.7
	Avoid crowded places	22	6
	Keep distance	66	18.1
	Stay indoors	92	25.0
	Do nothing	24	6.5
How ften do yo go to the hospital for medical check up	Twice or more in a year	59	17.9
	Once every year	56	17.0
	Once every two years	67	20.2
	Once or twice in 5 years	19	5.8
	Never in the last five years	64	19.3
	Others; not at all	65	19.7
Where do you go to when your sick	Health facility	324	88.3
	Pharmacy	74	20.2
	Traditional healer	33	8.9
	Pursue Other self-treatment options	49	13.3
What point would seek medical help from a hospital when had symptoms of TB	As soon I realise	259	70.6
	When self-treatment does not work	65	17.7
	TB signs and symptoms have lasted for 3 to 4 weeks	22	6
	I would not go to hospital	21	5.6

Source: Authors compilation obtained from the field survey, 2018.

#### Factors related to PAK of the participants

Logistic regression shows that gender (OR, 0.37; CI 0.21 - 0.69; p =0.001), age (OR, 1.16; CI 1.11 - 1.22; p < 0.001), ethnicity (OR, 2.36; CI 1.04 - 5.33; p = 0.039), and level of education (OR, 1.49; CI 1.25 - 1.77; p < 0.001) were the predictors for the knowledge of TB. The predictors identified for attitude related to TB were gender (OR, 10.76; CI 2.16 - 53.61; p = 0.004), level of education (OR, 0.63; CI 0.41 - 0.95; p= 0.028) and religion (OR, 0.32; CI 0.15 - 0.69; p = 0.003). In the case of practices related to TB, gender (OR= 10.76, 95% CI: 2.16-53.61; p= 0.004) and the level of education (OR=1.38, 95% CI: 1.02-1.88; p= 0.035) were the identified predictors.

#### Healthcare seeking behaviour for tuberculosis signs and symptoms

**Fig. 3:**
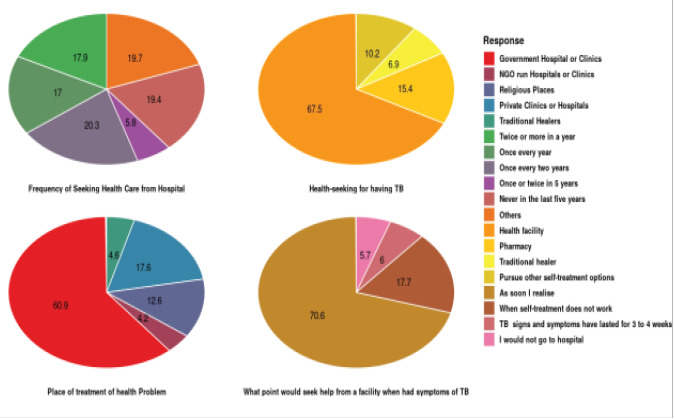
Healthcare seeking behaviour for tuberculosis signs and symptoms

The majority of the participants (86.7% n=318) indicated they would visit government hospitals when they find symptoms of TB, 6% (22) mentioned they would visit NGO health centres; also, 18.1% (66), 25% (92) and 6.5% (24) indicated they would visit religious places, private hospital or clinic and traditional healer respectively (Fig. 3). The results also showed that 20.2% (67) of the respondents visit the hospital once every two years, while 5.8% (19) visit the hospital once or twice in every 5 years. Regarding seeking healthcare when one realizes that he or she has contracted TB, 70.6% (259) of the respondents made it known that, they will seek medical attention while 5.6% (21) indicated that they will not go to the hospital at all.

## Discussion

We explored the underlying PAK of people in the PHAA-AND, about TB disease and the associated factors. The overall knowledge level of the participants about TB in the selected district was fairly high. This is not surprising since the majority of them have completed basic education. Most of them have the knowledge that TB is caused by bacteria. This outcome could probably be attributed to the efforts put up by the health system on public health education in the district. However, in the current study, a considerable proportion of the respondents indicated that TB is caused by sexual over indulgence, while others attributed it to witchcraft. These findings are in corroboration with previous studies where TB was attributed to other causes comprising witchcraft and sexual misconduct in the communities^15^. Additionally, a considerable proportion had the correct knowledge of symptoms of TB, including cough and weight loss. This contrasts with the results of a study done on predictors of tuberculosis knowledge in urban slums in Nigeria, where low knowledge on symptoms was very high^16^. Correct knowledge on transmission of TB was also high among the respondents as 85.6% knew that, TB was transmitted through aerosols of infected people. In line with good knowledge on the cause and transmission of TB, a high proportion of the respondents were familiar with preventive method of the disease. Similarly, the majority of the respondents, representing 96.5%, had good knowledge that tuberculosis is curable. This agrees with a study done by^17^ in Namibia, whereby over 90% of the respondents knew that TB is curable. Only a small proportion of the respondents had knowledge about DOTS. This knowledge gap implies that there is lack of proper information on the treatment system of TB in the community. The method of TB treatment is the DOTS and requires six months for treatment.^18^ When patients lack this information, it can result in defaulting, which further leads to MDR-TB.

In this study, 40% of the respondents were compassionate about TB patients and were willing to help them. Thus, a considerable proportion of the people in the district may have positive attitude towards TB patients in terms of extending help to them. In line with previous studies^19^; ^20^. Majority of the respondents 75.2% pointed that TB is very serious. This is a positive attitude towards TB in the district and indicates that people are likely to seek medical treatment if they contract the disease. Another positive attitude observed was that 62.8% of the respondents indicated that they will report to a health facility if they suspect to have TB. These positive attitudes could be related to the good knowledge the respondent have on TB. In terms of discrimination, the study showing TB carries more stigma than HIV, contrary to study conducted in the Sissala East of Ghana where HIV was seen to be more stigmatizing than TB.^21^ Nevertheless, 31.3% of the respondents indicated that TB disease carry less stigma than HIV AIDS and the minority of them said TB disease has the same stigma as the HIV/AIDS. These findings suggest that the respondents saw TB disease as a disease with more stigma, and this can affect case detection rate since those infected or with signs and symptoms might refuse to disclose it or seek treatment, thus possibly seeking treatment at inappropriate places.

A high proportion of the respondents indicating positive practice was consistent with other studies from^22^. Also, based on seeking health care when one realizes that he or she has contracted TB, significant majority 70.6% of the respondents indicated a positive action of seeking medical care. This concurs with the findings of a previous study done in the northern Ghana where higher proportion of the respondents stated they would seek healthcare attention immediately after observable symptoms of TB.^23^ It is important to point out that only 20% of the respondents visited a healthcare for regular medical check-up. This is likely to reflect the general situation in Ghana and requires serious attention.

It was found that gender, age, ethnicity and level of education have significant effect on the knowledge of TB disease among the respondents. The probability that males (77.9%) will be knowledgeable in TB is 0.37 times more than the females (54.9%) (OR, 0.37, CI; 0.21 - 0.69, p < 0.001). The probability that Akans will be knowledgeable in TB was 2.36 times higher compared to the non-Akans in this study. If the non-Akans could be traced and health education on TB be organized in their local dialect for them, it would help in TB health education among the people in the district. Level of education (OR, 1.49, CI; 1.25 - 1.77, p < 0.001) positively influenced the respondent's knowledge of TB disease. The odd ratio proves that as educational level goes up, the knowledge of TB disease increases by 1.49. This confirms findings of studies done elsewhere^24^;^25^. Age (OR, 1.16, CI; 1.11 - 1.22, p < 0.001) was significantly associated with the knowledge of TB disease. This could be that, people become more concern concerned about their health as they age. The study found that level of education was associated with attitude of the people in relation to the seriousness of TB disease (OR, 0.63, CI; 0.41-0.95, p=0.028). Similar results were found in a study conducted in Lesotho^26^, and possible reasons for this include educated people having better understanding of the disease and its consequences. Other predictors found are gender (OR, 10.76, CI; 2.16 - 53.61, p = 0.004) and religion (OR, 0.32, CI; 0.15-0.69, p=0.003). Practices towards TB prevention such as covering mouth and nose when coughing or sneezing was associated with gender and level of education (OR, 10.76, CI; 2.16 - 53.61, p = 0.004 and OR,1.38, CI; 1.02 - 1.88, p = 0.035). Again, this reflects the positive effect education has on positive practices related to TB and other disease.

## Conclusion and recommendations

The study population has good knowledge of TB, however, their knowledge is poor regarding TB treatment. Generally, the study population demonstrated a positive attitude to TB, as a high majority perceived the disease to be serious and were prepared to seek medical care on time. They also expressed good practices in relation to infection control of TB. For example, a high majority of the respondents (81.7%) indicated that mouth and nose should be covered during sneezing to avoid TB transmission from infected individuals. The study also concludes that the key predictors of TB knowledge of the study population are gender, level of education, age and ethnicity; the key factors associated with TB related attitudes are gender, level of education and religion; gender and level of education were the factors associated with practices towards TB prevention.

We recommend that there is the need for health education of the study population based on the gaps identified in their PAK related to tuberculosis. This is the responsibility of the Ghana health service. Further studies are needed on the PAK of other high burden diseases in the study community by public health practitioners and health researchers. There is the need for a community programme by healthcare workers, that would encourage residents to visit the hospital for regular medical examination of their health
